# Reactivation of Acute Retinal Necrosis following SARS-CoV-2 Infection

**DOI:** 10.1155/2021/7336488

**Published:** 2021-07-16

**Authors:** Monica P. Gonzalez, Radames Rios, Mariella Pappaterra, Miguel Hernandez, Allison Toledo, Carmen Santos, Andres Emanuelli, Shree K. Kurup, Armando L. Oliver

**Affiliations:** ^1^University of Puerto Rico, Medical Sciences Campus, Department of Ophthalmology, Paseo Dr. Jose Celso Barbosa, San Juan 00921, USA; ^2^Ponce Health Sciences University School of Medicine, 388 Zona Industrial Reparada 2, Ponce, PR 00716, USA; ^3^Emanuelli Research and Development Center, 452 Dr R Rivera Aulet Avenue, Suite 45 Arecibo, PR 00612-4368, USA; ^4^Department of Ophthalmology & Visual Sciences, Case Western Reserve University, University Hospitals Cleveland Medical Center, 11100 Euclid Avenue, Cleveland, OH 44106, USA

## Abstract

**Purpose:**

To report on a case of reactivation of acute retinal necrosis following SARS-CoV-2 infection.

**Methods:**

Observational case report. *Observations*. A 32-year-old female with a distant history of left retinal detachment secondary to necrotizing herpetic retinitis complained of right-eye vision loss, pain, redness, and photophobia. An ophthalmological examination revealed findings consistent with acute retinal necrosis of the right eye. A polymerase chain reaction (PCR) analysis of the right vitreous was positive for herpes simplex virus type 2 (HSV-2). A coronavirus disease 2019 (COVID-19) screening test using reverse transcriptase- (RT-) PCR was positive for SARS-CoV-2 RNA.

**Conclusions:**

Our case suggests that COVID-19 may cause a latent HSV infection to reactivate, causing contralateral involvement in patients with a prior history of HSV-associated acute retinal necrosis.

## 1. Introduction

Acute retinal necrosis (ARN) is a rare posterior uveitis syndrome characterized by rapidly progressive vision loss that may affect both immunocompetent and immunodeficient patients of any age [1, 2]. ARN is characterized by discrete foci of retinal necrosis that progress rapidly in a circumferential fashion, with associated occlusive arterial vasculopathy and significant inflammation of the posterior and anterior segments of the eye [1]. It is most commonly associated with the varicella-zoster virus (VZV) and herpes simplex viruses 1 and 2 (HSV-1 and HSV-2) [3]. Common sequelae of the disease include retinal breaks, detachments, and macular edema; patients with ARN tend to have a guarded visual prognosis [3, 4]. Among the most feared ARN complications is contralateral involvement, which may lead to significant visual disability and blindness [3]. In a case series by Lei et al., 25 out of 30 patients with ARN developed the disease in the contralateral eye, most commonly within months of the initial presentation; however, a delayed involvement of the fellow eye, occurring after several years, was also described in a small subset of patients [5]. Prompt treatment with systemic acyclovir substantially reduces the risk of bilateral involvement [6]. Although long-term antiviral therapy with acyclovir may prevent ARN involvement in the fellow eye, its role remains undefined [6]. For ARN, the duration of secondary antiviral prophylaxis has not been thoroughly assessed; however, some important factors to consider are immune status and comorbidities [1, 7].

Herpesviruses are a large family of DNA viruses with high prevalence in the population [8]. It is estimated that approximately 90% of adults worldwide are infected with HSV-1, HSV-2, or both [8]. The lytic (active) and latent stages of these viruses ensure that, once infected, an individual will remain so throughout his or her lifetime [8]. When the virus enters the latent stage, a balance of various factors, including the continuous response of the adaptive immune system, is required for the virus to stay dormant [9]. The impairment of CD8+ T cell suppression of viral lytic gene expression could lead to an HSV recurrence [9]. The reactivation of herpesvirus-related infections, specifically of herpes zoster dermatitis, in patients with the coronavirus disease 2019 (COVID-19) superinfection, has recently been described, yet a precise mechanism for the said reactivation has only been hypothesized [10, 11].

SARS-CoV-2 causes COVID-19, whose symptoms include mild to severe respiratory illness and sundry systemic manifestations [12]. After entering the human body, SARS-CoV-2 binds to the angiotensin-converting enzyme 2 protein present on the host's pulmonary epithelial cells. Viral entry and fusion are then followed by a robust immune response involving the adaptive and the innate immune systems. This immunological cascade has been described to cause immune system dysregulation and to cause decreased lymphocyte counts, along with decreasing CD3, CD4, and CD8 T lymphocytes [13, 14].

The first description of the ocular manifestations of COVID-19 was reported by Wu et al. in a case series of 38 patients in which ocular findings were present in about 31.5% of the patients [15]. Wu et al. described the ocular manifestations of COVID-19 as conjunctival hyperemia, chemosis, and epiphora [15]. Marinho et al. reported the optical coherence tomography findings of 12 symptomatic patients with polymerase chain reaction- (PCR-) proven COVID-19 infection [16]. In their report, they described the presence of multiple hyperreflective retinal lesions at the ganglion cell and inner plexiform layers and cotton wool spots and microhemorrhages on a fundus exam [16].

To our knowledge, no prior cases of ARN reactivation after SARS-CoV-2 infection have been reported. We hereby report a case of a 32-year-old Hispanic female with a prior history of ARN in her left eye who presented with contralateral ARN after having been infected with SARS-CoV-2.

## 2. Case Report

A 32-year-old Hispanic woman reported sudden vision loss in her right eye (OD), associated with a 1-week history of pain, redness, and photophobia. She had a history of ARN in the left eye (OS), which had occurred 3.5 years prior. She had received treatment with IV acyclovir for 2 weeks, followed by oral valacyclovir, which she had discontinued 1 year before the current presentation. Her left ARN had caused rhegmatogenous retinal detachment, which resulted in the loss of light perception, despite the best surgical efforts. Her past medical history and review of systems were otherwise unremarkable. She was sexually active with her husband and had no history of alcohol or illicit drug abuse.

The best-corrected visual acuity was finger counting at 5 feet in OD; she had no light perception in OS. The intraocular pressure was 10 mmHg in OD and 5 mmHg in OS. Her right pupil was round and reactive to light; the left pupil was corectopic and unreactive, and a left afferent pupillary defect was present; finally, the left eye had atrophic phthisis bulbi. A slit-lamp exam of her right eye revealed diffuse conjunctival injection, punctate epithelial erosions, Descemet folds, and granulomatous keratic precipitates in Arlt's triangle. The anterior chamber had 1+ cells and flare, and the lens had 1+ nuclear sclerosis.

**Figure 1:**
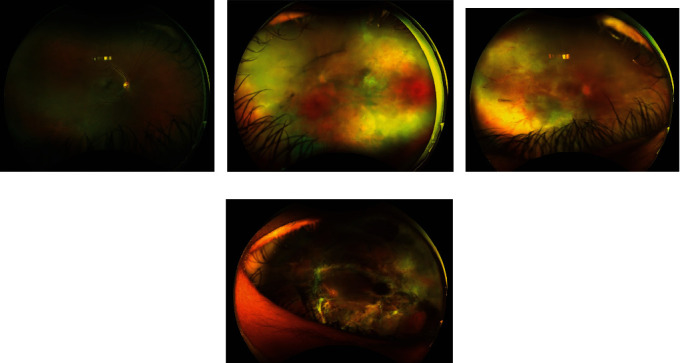
Ultra-widefield fundus photographs of the right (a, c) and left (b, d) eyes. Forty-three months prior to presentation, though the right eye remained uninvolved (a), the left eye developed extensive necrotizing retinitis involving over 80% of the retina. At presentation, the right eye (c) shows vitreous opacities, blurring of the disk margins, and retinal necrosis extending from 1 to 11 o'clock in zones 2 and 3 and diffusely in zone 1, sparing a small patch of the retina temporal to the optic disk. The left eye (d) has persistent retinal traction with fibrous tissue.

A dilated fundus exam of the right eye revealed 1+ vitritis, optic nerve edema, and retinal necrosis extending from 1 to 11 o'clock in zones 2 and 3 and in zone 1, sparing a small patch of the retina temporal to the optic disk (Figure 1(c)). The left eye revealed persistent retinal traction with fibrous tissue (Figure 1(d)). A fluorescein angiogram of the right eye revealed disk hyperfluorescence, macular and perivascular leakage, and severe peripheral capillary nonperfusion. The extraocular physical exam was unremarkable; in particular, she had normal vital signs, her lungs were clear to auscultation, her abdomen was soft to palpation, and her neurological status was unremarkable.

As her clinical findings were highly suggestive of ARN, she was admitted to the ward and treated with IV acyclovir (800 mg every 8 hours), topical prednisolone acetate 1% eye drops (4 times daily), and atropine sulfate 1% eye drops (3 times daily). A vitreous tap was performed, the resulting sample was sent for PCR analysis, and ganciclovir (2 mg/0.05 cc) was injected intravitreally. Forty-eight hours after commencing therapy, the infectious disease service switched her IV therapy to ganciclovir (380 mg IV every 12 hours).

Despite treatment, her clinical course continued to deteriorate, and after 4 days of therapy, she developed an inferior retinal detachment, which caused her visual acuity to drop to “hand motion.” The patient was scheduled for a pars plana vitrectomy with a silicone oil tamponade to repair the retinal detachment, and as part of the preoperative workup, a SARS-CoV-2 PCR test was ordered. The following day, the patient's visual acuity was “light perception,” and the partial retinal detachment had progressed to a total detachment.

Twenty-four hours later, the SARS-CoV-2 PCR test was reported as positive. No surgical management was pursued because of the patient's poor visual prognosis and positive SARS-CoV-2 test result. The patient continued to receive IV ganciclovir for 14 days. She was placed in an isolation room and received prophylactic anticoagulation with enoxaparin (40 mg subcutaneous every 12 hours) and iron supplementation to avoid COVID-19-associated complications. During her hospitalization, the patient had no COVID-19-associated symptoms and did not develop any other related complications.

The vitreous PCR test result was positive for HSV-2, confirming the diagnosis of HSV-2-associated ARN. Her serology results revealed positive HSV-1 IgG, HSV-2 IgG, CMV IgG, and VZV IgG. Toxoplasma antibodies (IgG and IgM), serum rapid plasma reagin, and human immunodeficiency virus testing were all negative. The vitreous PCR was negative for HSV-1.

## 3. Discussion

The nature of the interaction between SARS-CoV-2 and other herpesviruses, such as HSV-2, remains uncertain. Following infection with SARS-CoV-2, a series of hyperinflammatory events occur, leading to immune system dysregulation at the cellular and molecular levels [13]. Tartari et al. reported on 4 patients who developed cutaneous zoster while being hospitalized for COVID-19, all of whom had decreased CD3+ CD8+ lymphocytes before the onset of zoster [10]. Cutaneous zoster reactivation has also been described in patients with asymptomatic COVID-19 [11]. COVID-19 and VZV coinfection, presenting as a trigeminal neuropathy, have also been reported in a 39-year-old immunocompetent man with very few COVID-19 symptoms [17]. The altered immune state that occurs following SARS-CoV-2 infection may potentially serve as the gateway for latent HSV-2 reactivation.

The bilateral involvement of ARN usually occurs within months of the initial presentation [5]. In our case, the reactivation in the fellow eye occurred 4 years after the initial presentation. Although contralateral involvement seldom occurs several years after the initial episode, in our patient, it may have been triggered by the secondary immune vulnerability caused by a SARS-CoV-2 superinfection [5]. Despite the fact that our patient was clinically asymptomatic, we hypothesize that SARS-CoV-2 may have caused decreased peripheral CD3+ CD8+ T lymphocyte counts, thus inhibiting both her regulatory mechanisms and her protection against latent HSV [18].

Patients who present with acute or reactivated diseases associated with immune system dysfunction, such as ARN, may warrant an evaluation for possible SARS-CoV-2 infection, at least during the remainder of the pandemic. In addition, patients with a personal history of conditions such as ARN, where patients may be vulnerable to immune system deregulation, should remain vigilant for potential reactivations in the setting of a SARS-CoV-2 RNA superinfection.

Due to the low recurrence rate of ARN, long-term antiviral therapy may not be a mainstay of clinical practice [19]. However, antiviral treatment has been shown to reduce the risk of developing bilateral ARN [6]. Therefore, in patients who have a history of ARN in 1 eye, the benefits of preventing a recurrence of ARN in the contralateral eye may outweigh the risks associated with long-term antiviral therapy. Given the altered immune state caused by COVID-19, patients with a prior history of ARN or any other ocular HSV manifestation may be at increased risk of disease reactivation and, therefore, should consider secondary antiviral prophylaxis during SARS CoV-2 infection.

## 4. Conclusion

Our case suggests that ARN reactivation may occur following COVID-19 infection. Ophthalmologists should be aware of the predisposition that COVID-19's impact on the immune system may induce the reactivation of HSV-related diseases. Secondary prophylaxis with systemic antiherpetic medications should be considered for patients with a prior history of vision loss due to ARN, particularly if they are suffering from COVID-19.

## Data Availability

The manuscript does not have an affiliated dataset. It is a case report of a patient seen at the University of Puerto Rico School of Medicine affiliated hospitals and the Emanuelli Research and Development Center.
